# Expression Profiling of a Genetic Animal Model of Depression Reveals Novel Molecular Pathways Underlying Depressive-Like Behaviours

**DOI:** 10.1371/journal.pone.0012596

**Published:** 2010-09-07

**Authors:** Ekaterini Blaveri, Fiona Kelly, Alessandra Mallei, Kriss Harris, Adam Taylor, Juliet Reid, Maria Razzoli, Lucia Carboni, Chiara Piubelli, Laura Musazzi, Girogio Racagni, Aleksander Mathé, Maurizio Popoli, Enrico Domenici, Stewart Bates

**Affiliations:** 1 Medicines Research Centre, GlaxoSmithKline, Stevenage, United Kingdom; 2 Center of Neuropharmacology-Department of Pharmacological Sciences and Center of Excellence on Neurodegenerative Diseases, University of Milan, Milan, Italy; 3 Neurosciences CEDD, GlaxoSmithKline Medicines Research Centre, Verona, Italy; 4 Clinical Neuroscience–Psychiatry, Karolinska Insitutet, Huddinge Hospital, Stockholm, Sweden; 5 Instituto Di Ricoverio e Cura a Carattere Scientifico, San Giovanni di Dio-Fatebenefratelli, Brescia, Italy; 6 Cancer Research UK, London, United Kingdom; Max-Planck-Institut für Neurobiologie, Germany

## Abstract

**Background:**

The Flinders model is a validated genetic rat model of depression that exhibits a number of behavioural, neurochemical and pharmacological features consistent with those observed in human depression.

**Principal Findings:**

In this study we have used genome-wide microarray expression profiling of the hippocampus and prefrontal/frontal cortex of Flinders Depression Sensitive (FSL) and control Flinders Depression Resistant (FRL) lines to understand molecular basis for the differences between the two lines. We profiled two independent cohorts of Flinders animals derived from the same colony six months apart, each cohort statistically powered to allow independent as well as combined analysis. Using this approach, we were able to validate using real-time-PCR a core set of gene expression differences that showed statistical significance in each of the temporally distinct cohorts, representing consistently maintained features of the model. Small but statistically significant increases were confirmed for cholinergic (chrm2, chrna7) and serotonergic receptors (Htr1a, Htr2a) in FSL rats consistent with known neurochemical changes in the model. Much larger gene changes were validated in a number of novel genes as exemplified by TMEM176A, which showed 35-fold enrichment in the cortex and 30-fold enrichment in hippocampus of FRL animals relative to FSL.

**Conclusions:**

These data provide significant insights into the molecular differences underlying the Flinders model, and have potential relevance to broader depression research.

## Introduction

Major depressive disorder is a common disease, with a lifetime prevalence of up to 20% [1]. Although several animal models of depression have been developed, a model that replicates all of the aetiological factors causing depression in humans is currently lacking [2]. Flinders rats are a genetic model of depression, derived by selective breeding of Sprague-Dawley (SD) rats for their hypersensitivity or resistance to treatment with the anticholinesterase diisopropylfluorophosphate (DFP) [3]–[4] to derive Flinders Sensitive (FSL) and Flinders Resistant (FRL) lines [5]. The FSL rat shows many key behavioural features of depression in humans including a reduction in general activity, appetite and latency of REM sleep, immune abnormalities [3]–[6] and cholinergic hypersensitivity and serotonergic/dopaminergic abnormalities [7]. The usefulness of Flinders rats as a genetic model of some aspects of human depression is evident, however we have only an incomplete understanding of the molecular mechanisms underlying the behavioural abnormalities.

Despite its widespread adoption in other fields, transcriptional profiling has been employed only relatively infrequently in studies of human depression [8]–[12]. A number of studies reporting the use of gene expression profiling to study rodent models of depression have been seen including stress models [13]–[16], genetic susceptibility [17] and surgically induced models of depression [18]–[20]. Finally, a recent study [20] looked for consistency of gene expression changes in depression induced by pharmacological and surgical intervention. What has been particularly notable from these reports has been the lack of commonly regulated gene changes across different models and indeed within the same model across studies. There may be many reasons to account for these differences, either relating to the complexity of the models themselves, the power of the studies, the magnitude of the gene changes or the technical aspects of generating gene expression signatures.

In this study, genome-wide expression profiling of the hippocampus (HIP) and the prefrontal/frontal cortex (P/FC) of Flinders model was evaluated to identify the molecular and cellular pathways related to the pathophysiology of the depression-like phenotype in this model. The study was performed in two independent and temporally distinct cohorts of animals, with each cohort in the study well-powered to identify the subtle changes typical in these models. Using this novel well-powered approach, we were able to identify consistently maintained gene changes both within and across brain regions. The genes identified provide additional insights into the neurobiological processes underlying the behavioural abnormalities of Flinders Model, and molecular basis of the depressive phenotype.

## Results

### Study Design

Several gene expression studies on both human depression and rodent models of depression have clearly shown that brain transcriptional responses to depression or depression-like models are generally small in magnitude, variable in response and show little consistency between studies. At the outset of this study then, we decided to establish two independent cohorts of animals to allow us to identify gene expression differences that were consistently maintained across such temporally distinct cohorts. Based on power calculation from previous rodent studies and our expectation of low magnitude gene expression differences (typically below 2-fold in magnitude), the study was designed with at least 8 animals from each line in both cohorts. Following breeding, cohort one was established with 12 FRL and 9 FSL animals, while the second cohorts (6 months later) was made up of 10 FRL and 8 FSL animals.

### Behavioural Testing - Forced swim test

Before proceeding to microarray analysis, animals from each cohort were independently tested to monitor their responses to the forced swim test paradigm. Both cohorts of animals showed significant differences in response between FRL and FSL lines, FSL rats being significantly more immobile than FRL rats (immobility time: cohort 1: FSL 160.3±15.6 s; FRL 98.1±14.5s, ANOVA F(1,19) = 8.31, p-value for: 0.0095; cohort 2:FSL 99.8±23.1s; FRL: 41.0±7.9s, ANOVA F(1,15) = 6.37, p-value 0.023). Results across the two cohorts were combined and the statistically significant difference between lines was confirmed (F(1,35) = 15.10, p<0.001) despite a significant contribution to the observed variability due to the animal cohort component ((F1,35) = 14.10, p<0.001). These data confirm that there are clear behavioural differences between the FRL and FSL animals in both cohorts within our study.

### Gene Expression Profiling

Following the forced swim test animals were allowed 1 week to recover before samples were collected for gene expression profiling. The majority of available data show that most stress related parameters return to normal levels shortly after exposure to the forced swim test [21], [22], but we cannot rule out the possibility that some residual effects of the stress could contribute to the gene expression profiles. After the rest period, animals were sacrificed and both hippocampal (HIP) and prefrontal/frontal cortex (P/FC) samples harvested for RNA isolation. Efforts were made to eliminate all potential sources of variability (see materials and methods) in both animal handling, sample processing and data generation. While consistency was maintained within each cohort, reagents varied between cohorts, therefore any overlap in gene expression between cohorts is likely to represent biologically conserved differences.

### Analysis of Gene Expression Data

A mixed model ANOVA was fitted to the data to estimate the effects of rat line, cohort and labelling batch. Contrast analysis between factor levels were performed for each probeset to estimate the differences and measure the statistical significance. Due to the distinct expression signatures for each brain region, data for HIP and P/FC were analysed separately. In line with our expectations, the number of differentially regulated probesets was small, while individual changes were generally low in magnitude (with some notable exceptions). Broadly similar results were seen with both brain regions, although slightly more changes were seen in P/FC than in HIP. In order to favour biologically maintained gene expression changes, we filtered the data to identify genes that were significantly regulated (p≤0.05) in each of the two cohorts, rather than relying on overall statistical significance in the study. Although limiting the analysis to probesets showing statistical significance in both cohorts is rather conservative, we reasoned that it removes biases based on large response in any one cohort and favours the identification of biologically relevant expression changes.

**Figure 1:**
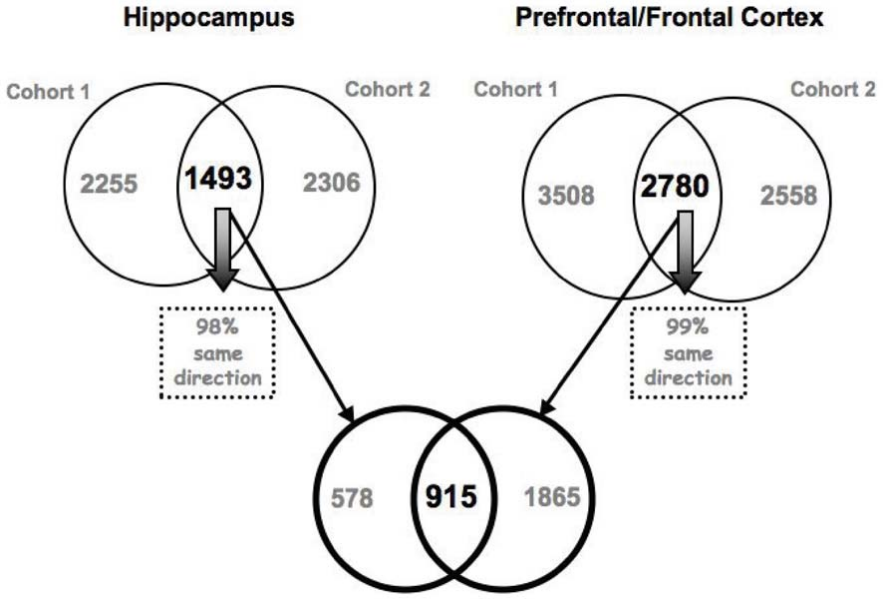
Gene expression summary. Venn diagram showing the number of probesets that were significantly regulated at p-value≤0.05 in HIP and P/FC from cohort 1 and cohort 2. Total number of probesets from each comparison are listed under each cohort, while numbers within the circles represent the breakdown of these figures with respect to each group: number of probesets in common highlighted in bold, while number of probesets specific to each cohort are coloured grey. Concordance in the directionality of response in the common changes is indicated by the arrows.

In HIP, 3,748 probesets were identified as differentially expressed between the FSL and FRL rats in the first cohort and 3,799 probe sets in the second cohort (at this p-value (<0.05) we would expect to find 1555 probesets changing by chance). However, when we looked between cohorts we found that approximately 40% of the probesets were significantly regulated in both cohorts (1,493 probesets (expect 78 by chance)) with a remarkable 98% concordance in the directionality of the gene changes (Figure 1 and supplementary tables S1 and S2). In P/FC there were 6,288 probesets identified as differentially expressed between the FSL and FRL rats in the first animal cohort and 5,338 probe sets in the second (at this p-value (<0.05) we would expect to find 1555 probesets changing by chance). Again in P/FC we found a more than 50% overlap (2,780 probesets (expect 78 by chance)) between cohorts, and 99% concordance in the directionality of responses (Figure 1).

Although not necessarily indicative of phenotypic relevance, we also looked at the overlap in gene expression profiles between HIP and P/FC. Of the 1,493 probesets in the HIP and 2,780 probesets in P/FC that were validated across both cohorts, 915 (or 61% of HIP differences) were differentially regulated across both brain regions in both cohorts (expect 4 by chance). Again, there was an almost complete concordance in the directionality of the response (99%) across all comparisons. A number of expression differences, however, did not show statistically significant changes in both brain regions, with 578 probesets significant only in the HIP and 1,865 only in P/FC (Figure 1).

These data suggest that the gene expression differences we have identified are consistently represented in this model across both cohorts and, in a large subset, also across brain regions. However, the magnitude of the differences was typically (although not exclusively) modest with the vast majority of changes less than 2-fold, in line with our expectations (Supplementary tables S1 and S2). All analyses were performed at the probeset level, which is a summary of the 11 individual pairs of oligos on the array, so while differential hybridisation is likely to reflect expression of transcripts in most cases, we cannot rule the possibility that sequence polymorphisms could account for some of the observed differences. Encouragingly, amongst the genes identified as changing consistently were some with known roles in cholinergic and serotonergic signalling, mechanisms that have previously been shown to be dysfunctional in the Flinders model (Supplementary table S3). These included the serotonin receptor 1a (Htr1a) and cholinergic receptors, muscarinic receptor 2 (Chrm2) and nicotinic receptor alpha 7 (Chrna7), which showed small but significantly higher expression in the P/FC of the FSL animals, while serotonin receptor 2a (Htr2a) was expressed at higher levels in the HIP of FSL animals. Small changes were also seen in the GABAergic receptors: GABA A receptor beta2 (Gabrb2) and GABA A receptor beta3 (Gabrb3). Although these changes were small in magnitude (1.1–1.4 fold), in all cases changes were assayed by multiple independent probesets and across both cohorts. Equally, no significant changes were seen in any other members of these receptors families, supporting the specificity of these small magnitude changes.

The largest gene expression differences were, however, seen in genes which for the most part have not previously been linked to depression-like phenotypes (see supplementary tables S1 and S2). The extremes of the changes were marked by 2.6 fold up-regulation and 6.0 fold down-regulation, although the magnitude of the changes rapidly falls off with less than 1% of the gene changes falling into this category (consistent with our premise at the outset). Amongst the genes showing the largest enrichment in FSL rats (relative to FRL) were the peroxisomal biogenesis factor 11b (Pex11b), Mutation Suppressor of sec4-8 (mss4) and a number of probesets representing novel transcripts. In contrast, genes including Carbonic Anhydrase III (CA3), Transmembrane protein 176A (TMEM176A), RNAse A Family 4 (RNase4) and a number of novel transcripts showed a reciprocal profile with reduced expression in FSL rats relative to FRL. The magnitude and statistical significance of these differences were remarkably well conserved across the two cohorts and even between the two brain regions (Supplementary data). A selection of genes showing larger gene changes, as well as the serotinergic/cholinergic/GABAergic genes were selected for further assessment using real-time PCR.

### Quantitative real-time PCR analysis of selected genes

**Figure 2:**
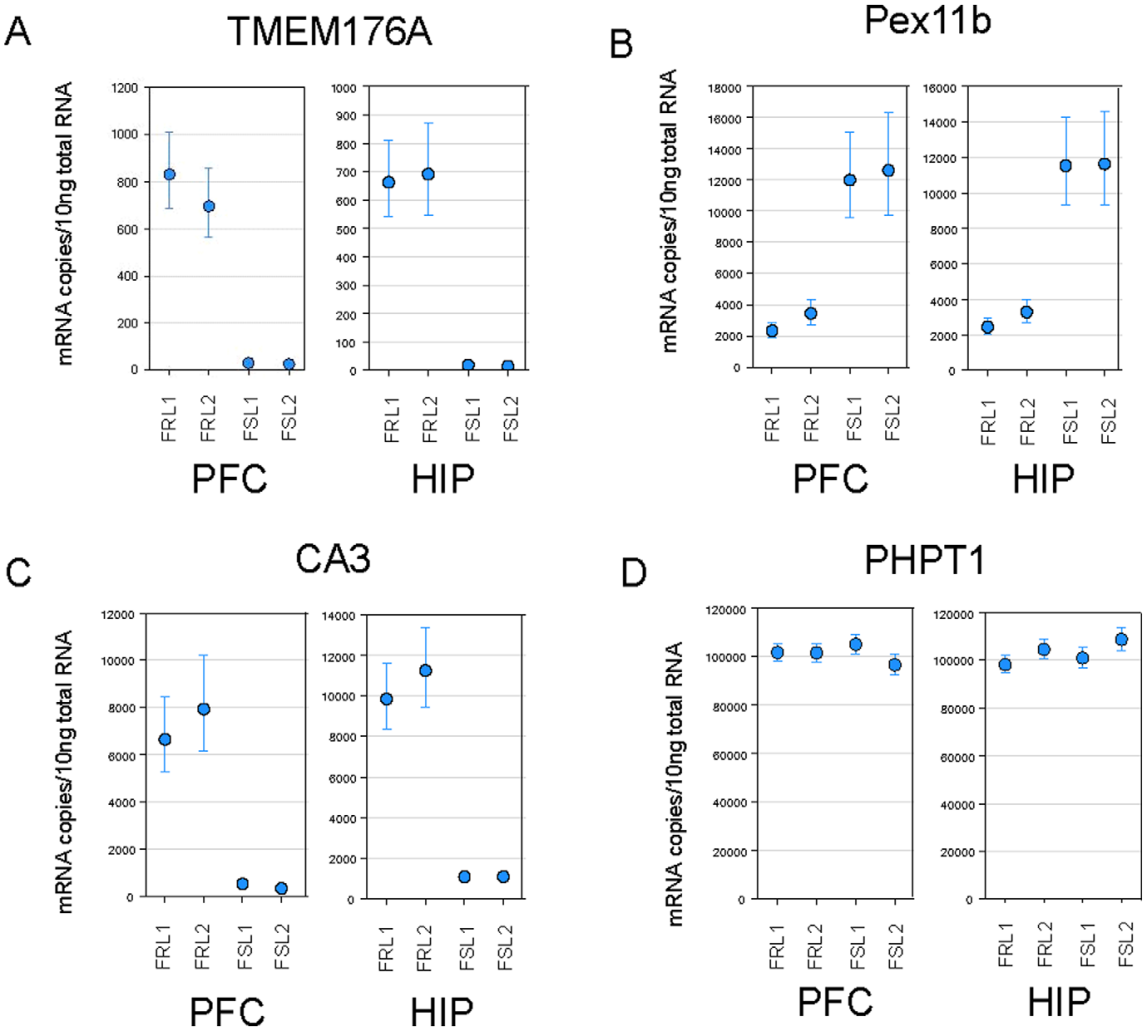
Examples of real-time PCR data. Group Least Squares Means were calculated in Array Studio and expressed as copy number/10ng of total RNA for FRL cohort 1 (FRL1), FRL cohort 2 (FRL2), FSL cohort 1 (FSL1) and FSL cohort 2 (FSL2) in P/FC and HIP. Data included for TMEM176A (A), Pex11b (B), CA3 (C) and invariant gene PHPT1 (D). Error bars represent 95% confidence intervals.

A total of 26 genes were selected for real-time PCR analysis, this included 19 genes that were predicted to show differential expression from the microarray data and seven selected as invariant or housekeeper genes. As the microarray data suggested some variation in expression of the typically-used housekeeper genes (notably GAPDH, β-actin and HPRT), the microarray data was used to select four additional genes (MRFAP1, RGD1310230, PHPT1_predicted, CDIPT) that showed invariant expression across the study. These invariant genes were run in addition to the traditional housekeeper genes in the real-time PCR. Small variations were indeed seen in the expression of the traditional housekeeper genes (see Table 2), while the 4 selected invariant genes showed consistent expression across both rat lines and in both cohorts (see Figure 2 for examples). Real-time PCR data were therefore normalised to these invariant genes and analysed using a similar statistical model to the microarray data.

The 19 genes selected for real-time PCR validation included a combination of genes that showed the most robust expression differences identified in the microarray analysis (Carbonic Anhydrase 3 (CA3), RGD1565398_predicted, Family with Sequence Similarity to 111 member A (FAM111A), Peroxisomal Biogenesis Factor 11 beta (Pex11b), Mutation Suppressor of Sec4-8 (mss4), Rho GTPase-activating Protein (GRIT), Interleukin Enhancer Binding Factor 3 (ILF3), Transmembrane Protein 176A (TMEM176A), RNAse Family 4 Protein (RNAse4) and 4 novel genes (1383058_at, 1392736_at, AI37236, AA859982), as well as number of genes that were predicted to show smaller differences but have previously been implicated in the pathophysiology of depression (Chrm2, Chrna7, Htr1a, Htr2a, Gabrb2 and Gabrb3: Supplementary Table S3).

The same mixed model analysis of variance was fitted to the real-time PCR data as was fitted to the microarray data with the addition of a covariate to correct for RNA loading, and each assay analysed separately in the model. A good concordance was seen between the microarray and real-time PCR data for most genes, with 17/19 genes showing statistically significant (p-value≤0.05) gene expression differences by real-time PCR in at least one brain region (see Tables 1 and 2). Two genes with robust microarray data (AI137236, and ILF3) failed to confirm using real-time PCR, and further work would be required to understand the reasons for this negative result. While the magnitude of the changes detected by real-time PCR were generally consistent with expectations from the microarray data, some genes showed significantly greater differential expression by real-time PCR than predicted by microarray, presumably reflecting the increased sensitivity (and therefore lower backgrounds) that can be achieved using real-time PCR. In agreement with the results from the microarray analysis, we were again able to confirm differential expression across both cohorts and in many cases also across brain regions (see Tables 1 and 2, and Figure 2). TMEM176A showed the largest differential expression difference, with 35 fold higher expression in cortex and 29-fold differential expression in the hippocampus of FRL animals as compared to FSL animals. Reciprocally Pex11b showed the largest FSL enriched expression with 4.5-fold increased expression in cortex and 4 fold-increased expression in hippocampus of FSL animals relative to FRL animals.

**Table 1:** P/FC Real-time PCR Summary.

Gene Name	Fold Change FSL vs FRL (combined)	p-value FSL vs FRL (combined)	Fold Change FSL vs FRL (cohort 1)	p-value FSL vs FRL (cohort 1)	Fold Change FSL vs FRL (cohort 2)	p-value FSL vs FRL (cohort 2)
TMEM176A	−40.2	**0.0E+00**	−35.1	**2.9E−37**	−48.0	**9.5E−36**
FAM111A	−19.7	**1.4E−08**	−32.2	**2.0E−07**	−10.2	**1.3E−03**
CA3	−16.9	**1.2E−35**	−12.6	**2.2E−24**	−24.0	**6.7E−28**
RNase4	−4.3	**5.3E−15**	−4.6	**2.1E−10**	−4.1	**3.4E−08**
RGD1565398_predicted	−3.3	**4.0E−32**	−3.3	**3.9E−24**	−3.2	**6.1E−20**
1392736_at	−1.6	**2.4E−13**	−1.6	**4.1E−10**	−1.5	**1.2E−06**
RICS_predicted	−1.1	3.0E−01	−1.1	3.9E−01	−1.0	5.9E−01
ILF3	1.0	4.0E−01	1.1	9.4E−02	−1.0	4.9E−01
Pex11b	4.4	**5.7E−22**	5.1	**1.3E−17**	3.7	**5.5E−11**
mss4	4.6	**0.0E+00**	4.5	**9.8E−45**	4.7	**3.6E−40**
1383058_at	2.1	**2.8E−28**	2.3	**4.6E−24**	1.9	**8.8E−15**
AA859982	1.3	**4.3E−14**	1.3	**1.5E−09**	1.3	**7.8E−08**
AI137236	−1.1	6.5E−02	−1.1	1.5E−01	−1.1	2.5E−01
Chrna7	1.1	**7.6E−03**	1.1	1.4E−01	1.1	**1.6E−02**
Chrm2	1.2	**9.5E−08**	1.2	**2.1E−06**	1.2	**3.1E−03**
Htr1a	1.3	**3.0E−09**	1.4	**2.0E−08**	1.2	**3.4E−03**
Htr2a	−1.0	8.8E−01	−1.1	3.4E−01	1.1	3.9E−01
Gabbr2	1.1	**1.4E−02**	1.0	3.8E−01	1.2	**5.9E−03**
Gabbr3	1.1	**3.6E−03**	1.1	**2.3E−03**	1.1	3.2E−01
GAPDH	1.1	**1.3E−06**	1.1	**2.3E−03**	1.2	**4.0E−05**
PPIA	1.0	1.4E−01	1.0	7.2E−01	1.1	6.2E−02
HPRT	1.2	**4.3E−10**	1.2	**4.0E−07**	1.2	**3.1E−05**
ACTB	1.2	**2.0E−07**	1.2	**2.4E−07**	1.1	**2.6E−02**

Table summarising output of mixed model analysis of variance analysis of the real-time PCR data for P/FC. Column 1 shows gene name; columns 2 and 3 show fold change and p-value respectively for the combined cohort analysis; column 4 and 5 show fold change and p-value respectively for cohort 1; columns 6 and 7 show fold change and p-value respectively for cohort 2.

**Table 2:** Hippocampus Real-time PCR Summary.

Gene Name	Fold Change FSL vs FRL (combined)	p-value FSL vs FRL (combined)	Fold Change FSL vs FRL (cohort 1)	p-value FSL vs FRL (cohort 1)	Fold Change FSL vs FRL (cohort 2)	p-value FSL vs FRL (cohort 2)
TMEM176A	−29.0	**0.0E+00**	−28.9	**8.4E−38**	−29.2	**2.0E−34**
FAM111A	−17.1	**2.1E−08**	−29.9	**3.6E−07**	−8.8	**1.5E−03**
CA3	−9.7	**2.2E−42**	−9.1	**3.9E−31**	−10.3	**4.1E−31**
RNase4	−3.5	**2.0E−20**	−3.6	**1.1E−13**	−3.4	**3.0E−12**
RGD1565398_predicted	−3.4	**3.4E−31**	−3.1	**1.5E−20**	−3.7	**1.7E−22**
1392736_at	−1.6	**8.6E−12**	−1.6	**1.3E−07**	−1.7	**6.2E−07**
RICS_predicted	−1.2	**2.0E−04**	−1.2	**1.3E−02**	−1.2	**2.4E−03**
ILF3	1.0	7.5E−01	1.0	4.7E−01	−1.0	7.3E−01
Pex11b	4.1	**2.0E−23**	4.7	**3.8E−18**	3.5	**1.0E−12**
mss4	4.3	**0.0E+00**	4.3	**0.0E+00**	4.2	**7.5E−43**
1383058_at	2.1	**2.5E−38**	2.1	**5.5E−28**	2.2	**4.7E−27**
AA859982	1.2	**9.7E−08**	1.2	**5.0E−04**	1.3	**1.3E−05**
AI137236	−1.2	**3.0E−04**	−1.2	**1.3E−03**	−1.1	6.4E−02
Chrna7	1.1	**2.6E−02**	1.1	**3.1E−02**	1.1	3.4E−01
Chrm2	1.1	7.8E−02	1.0	8.4E−01	1.2	**1.8E−02**
Htr1a	1.1	6.2E−02	1.1	4.3E−01	1.2	6.1E−02
Htr2a	1.3	**5.0E−06**	1.5	**1.1E−06**	1.2	8.8E−02
Gabbr2	1.0	7.3E−01	1.0	8.4E−01	1.0	7.8E−01
Gabbr3	1.0	9.5E−01	1.0	8.2E−01	−1.0	8.7E−01
GAPDH	1.1	**4.4E−02**	1.1	**3.4E−02**	1.0	4.8E−01
PPIA	1.0	5.8E−01	1.0	4.9E−01	1.0	9.6E−01
HPRT	1.1	**5.0E−02**	1.1	1.3E−01	1.1	2.2E−01
ACTB	1.1	**1.1E−01**	1.1	2.9E−01	1.1	2.2E−01

Table summarising output of mixed model analysis of variance analysis of the real-time PCR data for HIP. Column 1 shows gene name; columns 2 and 3 show fold change and p-value respectively for the combined cohort analysis; column 4 and 5 show fold change and p-value respectively for cohort 1; columns 6 and 7 show fold change and p-value respectively for cohort 2.

Focusing on the genes previously suggested to be involved in the pathophysiology of depression, we were able to confirm small but significant expression differences that were in-line with the microarray findings (Tables 1 and 2). Cholinergic receptors (Chrm2 and Chrna7), were significantly higher in expression levels in P/FC of the FSL compared to the FRL animals in both cohorts. Similar results were observed for the serotonin (Htr1a) and GABAergic receptors (Gababr2, Gababr3), again with a small but significant increase in expression in P/FC. Htr2a by contrast showed increased expression in HIP, again in line with expectation from the microarray data.

## Discussion

FSL/FRL rats were generated as a result of selective breeding of out-bred Sprague-Dawley rats for differences in the effects of the anticholinesterase agent diisopropylfluorophosphate (DFP) [23]. The FSL rats are more sensitive to DFP and cholinergic agonists than the counter-selected control FRL rats, a feature shared by depressed humans [24], which led to the original proposal of the FSL rats as an animal model of depression [25]. Consistent with the depression-like behaviour, FSL rats have been shown to display greater immobility in the forced swim test (FST) [25] compared to FRL rats. The Flinders rats therefore represent an attractive model to understand the molecular mechanisms underlying the depression-like phenotype, and this study represents the first report of genome-wide expression profiling of the model.

In this study we ran two, well statistically powered cohorts to allow independent analysis of each, while the combined analysis represents one of the largest reported studies to date for rodent depression models (22 FRL, 17 FSL). Profiling two brain regions from the same animals (HIP and P/FC) provides further biological validation as well as allowing the identification of a subset of gene expression differences that are consistently maintained across the two brain regions. The rigour of this approach is vouched for both by the observed overlap between cohorts (∼50% in P/FC, ∼40% in HIP) as well as the dramatic concordance in the directionality of response (approaching 100%). In addition to the comparison between FRL and FSL rats in this study, we also looked at the effects of maternal separation and anti-depressant drug treatment in other cohorts of the same animals (data not shown). The experimental approach and design mirrored those reported in this study, but unlike the robust gene expression changes noted in this study, we saw no statistically significant effects with either maternal separation or anti-depressant treatment across cohort or across brain region. These data suggest that the differences seen between FRL and FSL animals are both robust and reproducible, in a model where other readouts are not significant.

A total of 19 genes were selected for real-time PCR evaluation selected either based on the magnitude of the gene expression change predicted from the microarray analysis and/or previous linkage to the pathophysiology of depression, with the majority (17/19) confirming statistically significant differential expression. Novel gene TMEM176A had the largest differential expression in the real-time PCR analysis with almost undetectable expression in FSL rats, but very high levels in both the cortices and hippocampi of the FRL animals (35-fold differential expression in P/FC, 29-fold in hippocampus: see supplementary data and Figure 2). Genes showing a reciprocal expression pattern were also identified, notably Pex11b showed the highest level of elevated expression in the cortices and hippocampi of the FSL animals as compared to the FRL animals (approx 4-fold). These data clearly confirm the robustness of the microarray data, and show that dramatic gene expression differences exist between the FSL and FRL rat lines. To our knowledge these are some of the largest gene expression differences reported to date in a rodent model of depression.

A number of studies examining the neurochemical differences in the Flinders model have demonstrated substantial changes in both their cholinergic and serotonergic function in the FSL compared to the FRL rats [7], [26]–[28]. In line with these findings, we were able to show small but significant increased expression of the muscarinic 2 (Chrm2) and the nicotinic alpha 7 (Chrna7) receptors in P/FC of the FSL rats compared to the FRL animals (Table 1). These data are in line with the higher sensitivity to cholinergic agonists of FSL with respect to FRL animals [3], [5], [21], and consistent with a depression model mimicking the cholinergic supersensitivity observed in depressed patients [22], [29]. These data are also consistent with previous rodent profiling studies and human genetic studies that have implicated muscarinic 2 receptor in the aetiology of the depressed phenotype [30].

Significantly increased expression of the serotonin receptor 1a (Htr1a) in P/FC (Table 1) and the serotonin receptor 2a (Htr2a) in HIP (Table 2) was observed in the FSL line compared to the FRL line. These data are in-line with previous reports of increased Htr2a mRNA expression in the CA 2–3 region of the hippocampus of FSL using *in situ hybridization* [31], although other reports have noted little change in Htr1a [32]. The dysfunction of the serotonergic system in the FSL line is also documented by the increased tissue levels of 5-HT and 5-HIAA, that can be normalized by chronic antidepressant treatment [5], [7], [33]. In human depression, changes in the Htr1a and Htr2a receptors have been reported [34]–[39] and a 5-HT1A receptor polymorphism has been associated with depression [40].

A dysfunction of the GABA system has been reported in depressed patients [41]–[43] and in animal studies [40]. We identified small but significant increased expression levels of both Gabrb2 and Gabrb3 subunits of the GABA(A) receptor in P/FC of the FSL rats compared to the FRL rats (Table 1). To date the antidepressant-like effects of novel compounds that have a GABAergic mechanism have not been tested in the FSL rats.

Peroxisomal biogenesis factor 11b (Pex11b) showed the highest increased expression in both P/FC and HIP of the FSL animals, but has not been directly linked to depression previously. It is of interest that peroxisomal biogenesis disorders, which are genetic metabolic diseases with generalized, multiple, or single functional disturbances of the peroxisome, also present psychiatric symptomatology [44]–[46]. Oxidative stress has been suggested as a disease mechanism for major psychiatric disorders such as bipolar disorder, depression and anxiety disorders [47] with the most concrete evidence derived from studies conducted on schizophrenic patients.

A similar expression profile is seen with the Mutation Suppressor of Sec4-8 gene (mss4), which demonstrated significantly increased expression in the FSL animals in both brain regions and both cohorts. Mss4 is a guanine nucleotide exchange factor (also known as Rab Interacting Factor (RABIF)) which regulates the RAB family of small GTPases that are essential for intracellular vesicle transport and receptor recycling at the synapse. With important roles in neurotransmitter signalling and receptor trafficking, RAB proteins (particularly RAB3) have been implicated in various aspects of neuronal signalling, neurodegeneration and synaptic plasticity [48]. mss4 is likely to play a role in neurotransmitter release and synaptic plasticity, as it binds and stimulates GDP release from RAB3a, a key factor for BDNF-induced plasticity [49] and transmitter exocytosis [50]. mss4 was previously reported to be down-regulated in the hippocampus of anhedonic rats, this down-regulation being reversed by chronic antidepressant treatment [51].

CA3 is expressed at high levels in skeletal muscle but also at low levels in other tissues including brain. Our data show that CA3 is expressed at nearly 10-fold higher levels in both the cortices and hippocampi of the FRL animals as compared to FSL. While CA3 has not previously been implicated in depression or depression-like models, a number of reports have shown that SSRIs can activate carbonic anhydrase expression in the brain and suggest that carbonic anhydrase activation may contribute to the anti-depressive effects of these drugs [52]. It is tempting to speculate, therefore, whether overexpression of CA3 in FRL animals may mimic the effects seen with anti-depressant treatment and possibly contribute to the depression-resistant phenotype.

Many of the largest gene changes validated by RT-PCR were in novel or poorly studied transcripts (see Tables 1 and 2). For example, FAM111A and TMEM176A show the largest gene expression differences in the real-time PCR analysis with TMEM176A showing 35-fold differential regulation in the P/FC. Little is known or reported for these transcripts beyond their sequence, so much further work will be required before the significance of these relatively large transcriptional differences can be understood. It will also be interesting to understand to what extent these changes reflect the critical changes underpinning the depressive like phenotypes of this line, or merely the downstream responses to critical changes and whether the depressive phenotype could be reversed through intervention at these points. However, such substantial differential expression is highly unusual in psychiatric models in our experience, and represents a potentially significant finding deserving further work.

In conclusion, using robust experimental design and procedural standardisation, we have been able to identify biologically significant gene expression differences between FSL and FRL rats. Amongst the genes that were consistently differentially expressed were genes involved in cholinergic and serotinergic mechanisms which have previously been implicated in the aetiology of depression. Additionally we were able to identify a number of very significant expression changes in genes which have little or no previously linkage to depression. This represents a potentially novel insight in the molecular mechanisms underlying the Flinders phenotype and potentially depression more broadly.

## Materials and Methods

### Animals

The study was performed on adult male rats, bred at the Karolinska Institute. All animals were housed under standard housing conditions with access to food and water *ad libitum*.

Animal care and experimental procedures were conducted in compliance with the institutional guidelines and international laws and policies (European Communities Council Directive of 24 November 1986, 86/609/EEC).

### Behavioural Testing - Forced swim test

The behavioural procedure consisted of 2 exposures to a water tank that does not permit escape [53]. The water tank used was a transparent plastic tank, measuring 20 cm in diameter and 40 cm in height, containing 30 cm of fresh water at 25°C. Fresh water was used for each rat. During the first exposure, rats were placed into the tank, left there for 15 minutes and dried before they returned to their home cages. The second exposure occurred 24h afterwards and lasted 5 minutes during which rats behaviour was videotaped and subsequently scored by a trained experimenter blind to the animal experimental group. The rat was judged to be immobile when it floated passively, making only small movements to keep its nose above the surface. Immobility time, expressed as duration (s), was analysed utilizing mixed model analysis of variance (ANOVA) once their normal distribution was verified. Animal cohort was included in the analysis design as additional factor contributing to the observed variance. Significance level was set at p<0.05.

### Sample collection and RNA isolation

Animals were sacrificed by decapitation, Hippocampus (HIP) and the whole frontal lobe, referred to as prefrontal/frontal cortex (P/FC), were quickly excised on ice as previously described [54]–[55], and the right hemisphere was placed in RNA*later* (Qiagen, Inc., Valencia, CA, USA). Total RNA was isolated by homogenisation in TRIzol® Reagent (Invitrogen™ Life Technologies, Carlsbad, CA, USA), and RNA purified using the RNeasy® Mini Kit (Qiagen®, Inc., Valencia, CA, USA). RNA was quantified using spectrophotometric analysis and quality assessed using the Agilent 2100 bioanalyzer (Agilent Technologies, Palo Alto, CA, USA).

### RNA Amplification and Microarray Analysis

The standard Affymetrix One-Cycle Eukaryotic Target Labelling Assay protocol was used to generate cRNA probes that were subsequently hybridised to Affymetrix Rat Genome 230 2.0 GeneChips (http://media.affymetrix.com/support/technical/datasheets/rat230_2_datasheet.pdf). following manufacturer's guidelines (Affymetrix, Santa Clara, CA). The Affymetrix Rat Genome 230 2.0 GeneChip contains 31,000 probesets representing 28, 000 well substantiated rat genes. Samples were processed separately for each brain region with data generated in 2 batches for each cohort of animals. To avoid systematic errors, samples were processed in a pre-determined randomised order, with samples from each line equally distributed across batches. Single batches of reagents and a single lot of Affymetrix GeneChips were used for all samples within a cohort. A different randomisation scheme was used for microarray sample processing to that used for tissue collection. Microarray data was generated in a MIAME compliant format and raw data has been deposited in the GEO microarray database (Accession number GSE20388).

### Statistical Analysis of Microarray data

After scanning, all samples were found to be in the range of routine GeneChip quality assessment criteria and included in the data analysis. Signal intensities across all the arrays were normalised using Rosetta Resolver® Version 5.1 software (Rosetta Biosoftware, Seattle, WA, USA) [56] to adjust for technical variation across the data set. Only probesets that had normalised expression intensity greater than 30 in at least 50% of the samples in each rat line were included for further analysis. Intensities were then log transformed to ensure similar levels of variability across the range of signal intensities. A separate estimate of background variability for each probeset was estimated by fitting a statistical model that accounted for differences between rat lines, cohorts and batches (this technique is commonly called ANalysis Of VAriance, ANOVA).

For each probeset its estimate of background variability was then compared to the difference in the mean response of the rat lines to assess whether these differences were larger than we would expect by chance. This technique is commonly known as a post hoc comparison test. One of the outputs from such a test is a probability value (p-value) which is then used in the finally assessment of whether the mean difference is statistical significant. We defined everything with a p-value<0.05 as statistically significant. Our statistical analysis was performed in SAS® statistical software (SAS Institute, Cary, NC).

Functional analysis of the data was performed using the DAVID Bioinformatics Resources 2008 (http://david.abcc.ncifcrf.gov/home.jsp). Genes identified as differentially expressed between the FSL and FRL rats in each brain region from both animal cohorts were assessed for significant enrichment of particular biological processes using the terms of the fifth level of Gene Ontology (GO) (Supplementary Table S4).

### Real-time PCR Analysis

RNA samples were converted to cDNA using the High Capacity cDNA Archive Kit (Applied Biosystems, Foster City, CA). cDNA conversion was performed in a single batch, triplicate cDNA conversions for each RNA along with reverse-transcriptase minus controls for each sample. Real-time PCR results were generated using the 5′ nuclease assay (TaqMan) and the ABI 7900HT Sequence Detection System (Applied Biosystems, Foster City, CA). Each reaction included cDNA from 10ng of RNA, 900nM of each primer and 100nM of probe and Universal PCR Master Mix (Applied Biosystems, Foster City, CA). Abundance is calculated calculated for each real-time PCR assay separately using a standard curve generated using genomic DNA standards (all primers are designed to work with genomic DNA), and expressed as copies of RNA (after conversion to cDNA) per ng of total RNA. Assay sequence information is indicated in Supplementary Table S5. Primers were purchased from Sigma Genosys and FAM-TAMRA probes purchased from Biosearch.

### Statistical Analysis of Real-time PCR data

The same statistical modelling described in the analysis of the normalised microarray probeset data was performed on the real-time PCR data using ArrayStudio software (OmicSoft Corporation) with the difference that the rat line means used in the post hoc comparison tests were adjusted to account for differences in RNA loading. In our study we included four housekeeper genes that were identified by microarray analysis as being well expressed and invariant (MRFAP1, RGD1310230, PHPT1_predicted, CDIPT). Any changes in the expression of these genes represent differences in RNA loading between samples.

To summarise the changes across the three housekeepers we used Principal Components Analysis (PCA) to calculate a score for each sample. This score was then used to adjust the raw expression for each of the Taqman probes and thus remove the variability due to different levels of RNA loading. Bond et al gives more details on this method including showing how it is more efficient than traditional normalisation methods based on ratios [57]. As with the microarray data we defined anything from the post-hoc test with a p-value<0.05 as statistically significant. [57].

## Supporting Information

Table S1 Probesets with largest up-regulation in FSL. Table summarising probesets with the largest predicted up-regulation in FSL rats relative to FRL. Column 1 is Affymetrix probeset ID; column 2 is gene name; columns 3 and 4 are fold change and p-value respectively for combined analysis in PFC; columns 5 and 6 are fold change and p-value respectively for PFC in cohort 1; columns 7 and 8 are fold change and p-value respectively for PFC in cohort 2. Columns 9–14 are the equivalent HIP changes. Data is sorted based on fold change in PFC, and significant p-values are in bold. Grey boxes indicate genes selected for real-time PCR validation.
(0.06 MB PDF)

Table S2 Probesets with largest down-regulation in FSL. Table summarising probesets with the largest predicted down-regulation in FSL rats relative to FRL. Column 1 is Affymetrix probeset ID; column 2 is gene name; columns 3 and 4 are fold change and p-value respectively for combined analysis in PFC; columns 5 and 6 are fold change and p-value respectively for PFC in cohort 1; columns 7 and 8 are fold change and p-value respectively for PFC in cohort 2. Columns 9–14 are the equivalent HIP changes. Data is sorted based on fold change in PFC, and significant p-values are in bold. Grey boxes indicate genes selected for real-time PCR validation.
(0.07 MB PDF)

Table S3 Probesets for serotinergic, cholinergic and GABAergic receptors. Table summarising probesets for the serotinergic, cholinergic and GABAergic receptors showing significant changes in the model. Column 1 is Affymetrix probeset ID; column 2 is gene name; columns 3 and 4 are fold change and p-value respectively for combined analysis in PFC; columns 5 and 6 are fold change and p-value respectively for PFC in cohort 1; columns 7 and 8 are fold change and p-value respectively for PFC in cohort 2. Columns 9–14 are the equivalent HIP changes. Data is sorted based on fold change in PFC, and significant p-values are in bold.
(0.02 MB PDF)

Table S4 Gene ontology analysis of gene changes from hippocampus and PFC. Enriched Gene Ontology Biological Process annotation terms in the list of genes that were differentially expressed in the hippocampus (A) and PFC (B) of both study arms.
(0.25 MB PDF)

Table S5 Primer and probe sequence information for the real-time PCR assays.
(0.03 MB PDF)
