# Supplementary material for: Expression Profiling of a Genetic Animal Model of Depression Reveals Novel Molecular Pathways Underlying Depressive-Like Behaviours
Source: PLoS One. 2010 Sep 7;5(9):e12596. doi: 10.1371/journal.pone.0012596 (PMC2935375; doi:10.1371/journal.pone.0012596)
Supplement: Table S1 — Probesets with largest up-regulation in FSL. Table summarising probesets with the largest predicted up-regulation in FSL rats relative to FRL. Column 1 is Affymetrix probeset ID; column 2 is gene name; columns 3 and 4 are fold change and p-value respectively for combined analysis in PFC; columns 5 and 6 are fold change and p-value respectively for PFC in cohort 1; columns 7 and 8 are fold change and p-value respectively for PFC in cohort 2. Columns 9–14 are the equivalent HIP changes. Data is sorted based on fold change in PFC, and significant p-values are in bold. Grey boxes indicate genes selected for real-time PCR validation. (0.06 MB PDF) [file pone.0012596.s001.pdf]

Affy ID	Gene Name	Fold Change FSL vs FRL combined PFC	p-value FSL vs FRL combined PFC	Fold Change FSL vs FRL cohort 1 PFC	p-value FSL vs FRL cohort 1 PFC	Fold Change FSL vs FRL cohort 2 PFC	p-value FSL vs FRL cohort 2 PFC	Fold Change FSL vs FRL combined HIP	p-value FSL vs FRL combined HIP	Fold Change FSL vs FRL cohort 1 HIP	p-value FSL vs FRL cohort 1 HIP	Fold Change FSL vs FRL cohort 2 HIP	p-value FSL vs FRL cohort 2 HIP
1372923_at	Pex11b	2.6	2.8E-11	2.7	1.4E-08	2.4	5.1E-07	2.5	1.9E-10	2.8	2.5E-08	2.3	6.4E-06
1385871_at	AA859982	2.4	5.7E-18	2.5	2.9E-15	2.2	1.7E-12	2.2	1.8E-17	2.0	2.3E-12	2.4	4.2E-14
1383058_at	1383058_at	2.3	2.0E-19	2.5	2.0E-16	2.1	5.3E-13	2.5	4.9E-27	2.5	2.5E-22	2.5	1.1E-21
1382755_at	AI137236	2.2	4.2E-14	2.3	1.8E-11	2.0	2.6E-09	2.3	2.5E-18	2.2	1.5E-13	2.4	4.0E-14
1380507_at	1380507_at	2.2	2.4E-09	2.1	2.4E-06	2.2	2.4E-06	2.1	2.9E-09	1.9	6.6E-06	2.2	7.6E-07
1396743_at	1396743_at	2.1	2.4E-09	2.6	2.7E-08	1.8	2.9E-04	2.0	1.7E-09	2.5	5.1E-09	1.6	8.1E-04
1393410_at	Cntnap2_predicted	2.0	2.9E-08	2.0	7.9E-06	2.0	1.7E-05	1.6	1.7E-05	1.6	1.3E-03	1.7	1.1E-03
1380651_at	Rnf6_predicted	2.0	5.8E-08	1.6	1.3E-03	2.4	3.1E-07	1.7	5.0E-06	1.7	5.8E-04	1.8	4.2E-04
1379571_at	Pkp4_predicted	1.9	2.4E-06	2.1	5.1E-05	1.7	1.8E-03	2.0	1.6E-06	2.0	1.5E-04	2.0	3.7E-04
1394477_at	1394477_at	1.8	9.2E-10	1.8	1.1E-06	1.9	1.3E-06	1.7	8.7E-09	1.6	1.1E-05	1.7	3.4E-06
1369962_at	Atic	1.8	1.5E-08	2.0	4.5E-07	1.6	1.4E-04	1.7	2.4E-07	1.8	1.2E-05	1.6	3.0E-04
1380900_at	1380900_at	1.8	3.1E-14	1.9	2.3E-11	1.8	1.3E-09	1.8	2.5E-17	1.8	8.3E-14	1.8	2.9E-12
1376745_at	mss4	1.8	2.3E-17	1.7	9.0E-13	1.9	8.0E-14	1.7	2.4E-16	1.5	1.8E-09	2.0	4.8E-15
1385035_at	Usp12_predicted	1.8	7.9E-06	1.5	7.6E-03	2.1	4.7E-05	1.8	6.3E-07	1.7	2.4E-04	1.9	5.6E-05
1383298_at	RGD1310444_predicted	1.7	3.5E-08	1.9	1.3E-06	1.6	1.7E-04	1.9	4.2E-08	1.6	1.6E-04	2.1	1.4E-06
1390605_at	Cdh11	1.7	1.4E-08	1.4	8.8E-04	2.0	5.3E-08	1.7	3.7E-11	1.5	2.3E-06	1.9	3.0E-09
1397516_at	Alg2_predicted	1.7	2.2E-05	1.7	8.6E-04	1.7	1.9E-03	1.4	1.0E-04	1.3	9.9E-03	1.5	1.1E-03
1369773_at	Bmp3	1.6	1.6E-05	1.6	1.2E-03	1.7	8.3E-04	1.8	1.1E-06	2.1	4.1E-06	1.5	8.0E-03
1391564_at	LOC363022	1.6	5.3E-08	1.6	2.9E-05	1.7	1.1E-05	1.4	1.1E-05	1.4	8.4E-04	1.5	7.8E-04
1368379_at	Scarb2	1.6	5.6E-07	1.5	3.3E-04	1.7	3.2E-05	1.5	2.6E-07	1.6	8.3E-06	1.4	5.0E-04
1377422_at	Mtmr1_predicted	1.6	2.3E-05	1.6	9.2E-04	1.6	2.4E-03	1.5	1.5E-06	1.5	7.8E-05	1.4	7.8E-04
1377424_at	Dutp	1.6	4.4E-08	1.6	9.7E-06	1.6	4.5E-05	1.4	2.6E-05	1.4	4.8E-03	1.5	4.4E-04
1394693_at	GB:AB014881	1.6	9.1E-07	1.8	3.1E-06	1.4	8.8E-03	1.5	1.1E-06	1.6	5.7E-06	1.3	6.6E-03
1392171_at	Chi3l1	1.6	1.2E-09	1.7	3.6E-08	1.4	7.4E-05	1.7	2.3E-15	1.6	7.5E-11	1.7	7.7E-12
1384783_at	RGD:628712	1.6	4.7E-07	1.7	6.7E-06	1.4	1.4E-03	1.5	3.3E-07	1.6	5.0E-06	1.4	1.3E-03
1393716_at	LOC502421	1.5	8.5E-11	1.7	2.0E-09	1.4	1.2E-05	1.3	2.2E-05	1.3	9.3E-04	1.2	2.2E-03
1380164_at	Tk2_predicted	1.5	3.0E-07	1.5	1.4E-04	1.6	2.8E-05	1.5	2.8E-08	1.5	3.7E-06	1.4	5.4E-05
1371749_at	RGD1306001_predicted	1.5	5.7E-10	1.6	2.7E-08	1.4	3.3E-05	1.5	6.3E-09	1.5	1.2E-06	1.5	2.3E-05
1393783_at	Cntnap2_predicted	1.5	1.6E-07	1.6	2.0E-06	1.4	1.1E-03	1.3	1.3E-04	1.3	5.5E-03	1.3	3.4E-03
1398382_at	Mak3_predicted	1.5	1.4E-06	1.5	6.7E-05	1.5	6.7E-04	1.4	8.8E-06	1.4	9.7E-04	1.4	5.9E-04
1368883_at	Nov	1.5	1.6E-07	1.5	2.3E-05	1.5	9.2E-05	1.5	3.6E-06	1.5	4.1E-04	1.5	4.7E-04
1395453_at	LOC362398	1.5	1.7E-05	1.4	3.0E-03	1.5	3.8E-04	1.3	6.3E-06	1.2	6.0E-03	1.5	4.1E-05
1381906_at	Tpd52_predicted	1.5	1.3E-07	1.4	3.4E-04	1.6	4.6E-06	1.4	8.0E-06	1.3	3.6E-03	1.6	1.1E-04
1372647_at	Prelp	1.5	1.7E-08	1.6	7.9E-08	1.3	1.6E-03	1.6	7.6E-11	1.7	1.3E-08	1.5	2.9E-06
