# Supplementary material for: Expression Profiling of a Genetic Animal Model of Depression Reveals Novel Molecular Pathways Underlying Depressive-Like Behaviours
Source: PLoS One. 2010 Sep 7;5(9):e12596. doi: 10.1371/journal.pone.0012596 (PMC2935375; doi:10.1371/journal.pone.0012596)
Supplement: Table S2 — Probesets with largest down-regulation in FSL. Table summarising probesets with the largest predicted down-regulation in FSL rats relative to FRL. Column 1 is Affymetrix probeset ID; column 2 is gene name; columns 3 and 4 are fold change and p-value respectively for combined analysis in PFC; columns 5 and 6 are fold change and p-value respectively for PFC in cohort 1; columns 7 and 8 are fold change and p-value respectively for PFC in cohort 2. Columns 9–14 are the equivalent HIP changes. Data is sorted based on fold change in PFC, and significant p-values are in bold. Grey boxes indicate genes selected for real-time PCR validation. (0.07 MB PDF) [file pone.0012596.s002.pdf]

Affy ID	Gene Name	Fold Change FSL vs FRL combined PFC	p-value FSL vs FRL combined PFC	Fold Change FSL vs FRL cohort 1 PFC	p-value FSL vs FRL cohort 1 PFC	Fold Change FSL vs FRL cohort 2 PFC	p-value FSL vs FRL cohort 2 PFC	Fold Change FSL vs FRL combined HIP	p-value FSL vs FRL combined HIP	Fold Change FSL vs FRL cohort 1 HIP	p-value FSL vs FRL cohort 1 HIP	Fold Change FSL vs FRL cohort 2 HIP	p-value FSL vs FRL cohort 2 HIP
1371970_at	FAM111A	-5.4	**2.21E-10**	-8.1	**2.94E-09**	-3.6	**8.64E-05**	-3.9	**2.46E-10**	-5.1	**6.21E-09**	-2.9	**4.47E-05**
1371475_at	RNase4	-4.7	**1.89E-06**	-4.1	**4.83E-04**	-5.4	**1.56E-04**	-3.3	**2.56E-07**	-3.1	**1.11E-04**	-3.6	**4.14E-05**
1367896_at	CA3	-4.5	**4.10E-09**	-3.6	**2.17E-05**	-5.7	**5.26E-07**	-4.2	**3.23E-10**	-3.8	**1.10E-06**	-4.7	**2.44E-07**
1371349_at	RGD1565398_predicted	-4.4	**3.48E-18**	-4.9	**2.83E-15**	-3.9	**8.02E-13**	-2.2	**4.49E-22**	-2.2	**2.16E-17**	-2.3	**2.67E-17**
1387366_at	ILF3	-3.7	**2.23E-25**	-4.1	**2.12E-22**	-3.4	**1.99E-19**	-4.3	**9.99E-26**	-4.7	**3.48E-22**	-4.0	**6.34E-20**
1391985_at	Nrip3_predicted	-3.7	**9.19E-13**	-3.8	**1.93E-09**	-3.7	**1.43E-08**	-6.0	**1.34E-15**	-6.1	**7.07E-12**	-6.0	**4.11E-11**
1368943_at	Rnase4	-3.4	**7.19E-07**	-2.8	**6.44E-04**	-4.1	**2.80E-05**	-2.6	**7.76E-08**	-2.5	**3.86E-05**	-2.7	**2.39E-05**
1383393_at	RGD1309786_predicted	-3.2	**3.87E-13**	-3.4	**3.39E-10**	-3.1	**7.59E-09**	-3.9	**1.48E-12**	-3.3	**3.69E-08**	-4.5	**1.07E-09**
1386977_at	CA3	-2.8	**1.63E-09**	-2.7	**1.28E-06**	-2.8	**2.63E-06**	-2.8	**4.35E-10**	-2.8	**4.76E-07**	-2.9	**7.89E-07**
1392736_at	1392736_at	-2.7	**1.09E-14**	-2.9	**5.83E-12**	-2.5	**7.05E-10**	-2.9	**5.72E-13**	-2.9	**7.04E-10**	-2.8	**1.15E-08**
1380094_a_at	Zfp212_predicted	-2.7	**5.97E-18**	-3.2	**8.63E-16**	-2.2	**1.02E-10**	-2.7	**2.54E-24**	-3.0	**1.88E-21**	-2.3	**6.18E-17**
1369663_at	Ephx2	-2.6	**6.52E-13**	-2.4	**1.22E-08**	-2.9	**1.11E-09**	-3.4	**8.12E-13**	-4.0	**1.46E-10**	-2.9	**2.18E-07**
1375958_at	1375958_at	-2.5	**3.80E-09**	-2.2	**1.68E-05**	-2.9	**3.87E-07**	-2.5	**5.16E-11**	-2.0	**6.94E-06**	-3.1	**1.68E-09**
1377061_at	RICS_predicted	-2.4	**1.39E-12**	-3.0	**2.52E-11**	-2.0	**4.71E-06**	-2.6	**3.16E-10**	-2.9	**3.66E-08**	-2.4	**9.42E-06**
1380577_at	Abcg2	-2.3	**2.19E-10**	-2.1	**1.10E-06**	-2.4	**1.31E-07**	-2.0	**2.35E-09**	-1.7	**4.46E-05**	-2.3	**9.38E-08**
1370995_at	Pou2f1	-2.2	**2.22E-08**	-2.0	**3.55E-05**	-2.4	**4.04E-06**	-1.9	**5.71E-09**	-1.8	**2.06E-05**	-2.1	**9.24E-07**
1396344_at	1396344_at	-2.2	**3.45E-09**	-2.1	**4.27E-06**	-2.3	**2.32E-06**	-2.5	**1.06E-12**	-2.5	**3.18E-09**	-2.6	**1.06E-08**
1396846_at	Grik2	-2.2	**4.24E-13**	-1.9	**2.08E-08**	-2.4	**1.18E-10**	-3.3	**2.45E-13**	-3.0	**2.69E-09**	-3.6	**4.47E-10**
1389275_at	Sh3bp1_predicted	-2.1	**7.59E-10**	-1.6	**2.19E-04**	-2.7	**3.36E-09**	-1.6	**1.12E-09**	-1.6	**2.01E-06**	-1.7	**4.89E-07**
1380332_at	1380332_at	-2.1	**6.87E-09**	-2.2	**8.67E-07**	-1.9	**3.68E-05**	-2.1	**5.65E-11**	-2.3	**8.22E-09**	-1.9	**2.78E-06**
1393302_at	TMEM176A	-2.0	**4.17E-12**	-2.2	**6.86E-10**	-1.9	**6.27E-07**	-3.4	**2.71E-13**	-3.6	**3.15E-10**	-3.2	**1.29E-08**
1386855_at	GB:AA900968	-2.0	**2.92E-06**	-1.8	**1.32E-03**	-2.2	**9.28E-05**	-2.1	**7.44E-06**	-2.2	**3.10E-04**	-2.0	**1.65E-03**
1398445_at	1398445_at	-2.0	**2.10E-08**	-1.6	**4.11E-04**	-2.5	**1.35E-07**	-2.0	**1.58E-09**	-1.7	**7.70E-05**	-2.4	**2.40E-08**
1368826_at	Comt	-2.0	**6.02E-06**	-1.8	**2.31E-03**	-2.3	**1.01E-04**	-1.9	**5.78E-05**	-1.7	**5.63E-03**	-2.1	**1.05E-03**
1373204_at	TMEM176A	-1.9	**3.99E-12**	-1.9	**7.91E-09**	-1.9	**3.72E-08**	-1.6	**9.91E-12**	-1.5	**3.86E-08**	-1.6	**1.72E-08**
1376129_at	GB:BF393567	-1.9	**5.36E-07**	-1.8	**1.47E-04**	-1.9	**7.19E-05**	-2.1	**6.70E-09**	-2.3	**3.42E-07**	-1.9	**8.58E-05**
1369149_at	Limk1	-1.9	**1.80E-08**	-2.1	**3.46E-07**	-1.6	**2.47E-04**	-2.2	**1.09E-08**	-1.8	**1.91E-04**	-2.7	**2.16E-07**
1392381_at	1392381_at	-1.8	**5.91E-07**	-1.8	**1.74E-04**	-1.9	**9.05E-05**	-1.6	**2.75E-05**	-1.4	**8.75E-03**	-1.8	**2.25E-04**
1394160_at	Tmem2_predicted	-1.8	**1.11E-08**	-2.0	**4.24E-07**	-1.7	**1.59E-04**	-1.8	**1.19E-06**	-1.8	**1.07E-04**	-1.8	**4.08E-04**
1388654_at	Mrpl52_predicted	-1.8	**1.85E-07**	-1.8	**5.41E-05**	-1.9	**4.44E-05**	-1.3	**1.02E-04**	-1.3	**2.50E-03**	-1.3	**5.91E-03**
1398255_at	Slc15a2	-1.8	**6.44E-10**	-2.0	**2.32E-08**	-1.6	**2.35E-05**	-1.4	**3.14E-06**	-1.3	**9.16E-04**	-1.5	**1.23E-04**
1367811_at	Phgdh	-1.8	**2.59E-10**	-1.7	**4.87E-07**	-1.8	**1.95E-07**	-1.8	**2.92E-14**	-1.8	**9.16E-11**	-1.8	**9.79E-10**
1370234_at	Fn1	-1.8	**1.26E-12**	-1.7	**1.97E-08**	-1.9	**2.00E-09**	-1.6	**4.39E-11**	-1.4	**1.27E-05**	-1.8	**8.32E-10**
1373571_at	Rtn3	-1.8	**8.58E-19**	-1.6	**2.56E-13**	-1.9	**1.25E-15**	-1.7	**1.79E-20**	-1.6	**3.30E-14**	-1.9	**8.82E-18**
