# Supplementary material for: Expression Profiling of a Genetic Animal Model of Depression Reveals Novel Molecular Pathways Underlying Depressive-Like Behaviours
Source: PLoS One. 2010 Sep 7;5(9):e12596. doi: 10.1371/journal.pone.0012596 (PMC2935375; doi:10.1371/journal.pone.0012596)
Supplement: Table S3 — Probesets for serotinergic, cholinergic and GABAergic receptors. Table summarising probesets for the serotinergic, cholinergic and GABAergic receptors showing significant changes in the model. Column 1 is Affymetrix probeset ID; column 2 is gene name; columns 3 and 4 are fold change and p-value respectively for combined analysis in PFC; columns 5 and 6 are fold change and p-value respectively for PFC in cohort 1; columns 7 and 8 are fold change and p-value respectively for PFC in cohort 2. Columns 9–14 are the equivalent HIP changes. Data is sorted based on fold change in PFC, and significant p-values are in bold. (0.02 MB PDF) [file pone.0012596.s003.pdf]

Affy ID	Gene Name	Fold Change FSL vs FRL combined PFC	p-value FSL vs FRL combined PFC	Fold Change FSL vs FRL cohort 1 PFC	p-value FSL vs FRL cohort 1 PFC	Fold Change FSL vs FRL cohort 2 PFC	p-value FSL vs FRL cohort 2 PFC	Fold Change FSL vs FRL combined HIP	p-value FSL vs FRL combined HIP	Fold Change FSL vs FRL cohort 1 HIP	p-value FSL vs FRL cohort 1 HIP	Fold Change FSL vs FRL cohort 2 HIP	p-value FSL vs FRL cohort 2 HIP
1369809_at	Htr1a	1.3	**2.6E-05**	1.4	**2.0E-04**	1.3	**1.1E-02**	1.2	**5.4E-05**	1.2	**1.5E-02**	1.3	**2.7E-04**
1395089_at	Htr1a	1.2	**6.5E-06**	1.2	**1.7E-04**	1.2	**2.0E-03**	1.1	**1.3E-02**	1.0	9.1E-01	1.3	**7.5E-04**
1376458_at	Htr1a	1.1	**2.2E-03**	1.1	6.3E-02	1.1	**7.9E-03**	1.1	9.0E-02	1.0	9.1E-01	1.3	**2.0E-02**
1369124_at	Htr2a	1.2	**2.7E-02**	1.1	2.0E-01	1.2	5.3E-02	1.2	**1.7E-03**	1.2	**9.9E-03**	1.2	**4.6E-02**
1369125_at	Htr2a	1.2	**7.8E-03**	1.2	9.8E-02	1.3	**2.5E-02**	1.4	**4.2E-04**	1.5	**2.6E-03**	1.3	**3.0E-02**
1394517_at	Chrm2	1.2	**1.0E-06**	1.3	**1.1E-05**	1.2	**2.6E-03**	1.1	9.2E-02	1.0	7.5E-01	1.2	**3.5E-02**
1369245_at	Chrm2	1.1	**3.0E-02**	1.1	1.5E-01	1.1	8.8E-02	1.1	1.5E-01	1.0	6.4E-01	1.2	1.1E-01
1395429_at	Chrna7	1.1	**1.0E-02**	1.2	**4.1E-02**	1.1	9.5E-02	1.1	1.1E-01	1.2	1.1E-01	1.1	5.2E-01
1387419_at	Chrna7	1.2	**2.3E-04**	1.2	**1.3E-02**	1.2	**2.3E-03**	1.2	**9.1E-05**	1.2	**1.6E-03**	1.2	**7.1E-03**
1369818_at	Gabrb2	1.4	**1.7E-04**	1.3	**1.5E-02**	1.5	**1.3E-03**	1.1	3.7E-01	1.0	9.7E-01	1.2	2.1E-01
1369903_at	Gabrb3	1.4	**8.5E-06**	1.3	**1.5E-03**	1.4	**2.6E-04**	1.3	**2.9E-03**	1.2	1.3E-01	1.4	**4.4E-03**
1382132_at	Gabrb3	1.1	**5.0E-04**	1.1	**2.7E-03**	1.1	**3.8E-02**	1.0	**2.8E-02**	1.0	1.3E-01	1.0	9.9E-02
1378391_at	Gabrb3	1.1	**2.3E-02**	1.1	3.0E-01	1.1	**2.1E-02**	1.1	5.1E-02	1.0	4.9E-01	1.1	**3.3E-02**
