# Supplementary material for: Expression Profiling of a Genetic Animal Model of Depression Reveals Novel Molecular Pathways Underlying Depressive-Like Behaviours
Source: PLoS One. 2010 Sep 7;5(9):e12596. doi: 10.1371/journal.pone.0012596 (PMC2935375; doi:10.1371/journal.pone.0012596)
Supplement: Table S4 — Gene ontology analysis of gene changes from hippocampus and PFC. Enriched Gene Ontology Biological Process annotation terms in the list of genes that were differentially expressed in the hippocampus (A) and PFC (B) of both study arms. (0.25 MB PDF) [file pone.0012596.s004.pdf]

A

GOTERM_BP_5	Count	%	P-Value	FDR
synaptic vesicle transport	15	1.1	0.000	0.1
exocytosis	23	1.6	0.000	0.2
synaptic transmission	58	4.1	0.000	0.4
intracellular transport	81	5.7	0.000	0.4
secretory pathway	41	2.9	0.000	0.5
regulation of neurotransmitter levels	27	1.9	0.000	0.5
secretion by cell	46	3.2	0.000	0.6
neuron development	36	2.5	0.000	0.6
catecholamine metabolic process	11	0.8	0.001	1.5
microtubule-based process	30	2.1	0.001	1.9
cellular morphogenesis during differentiation	30	2.1	0.002	2.6
neuron differentiation	42	3	0.002	2.8
neurite morphogenesis	28	2	0.002	3.3
cell part morphogenesis	36	2.5	0.002	4.2
cell projection organization and biogenesis	36	2.5	0.002	4.2
cell projection morphogenesis	36	2.5	0.002	4.2
monosaccharide catabolic process	15	1.1	0.003	4.9
cell redox homeostasis	11	0.8	0.003	5.1
protein transport	67	4.7	0.003	5.3
cytoskeleton-dependent intracellular transport	18	1.3	0.004	6.3
microtubule-based movement	16	1.1	0.004	6.7
neurogenesis	49	3.4	0.004	7.5
dopamine metabolic process	8	0.6	0.006	10.2
modification-dependent macromolecule catabolic process	21	1.5	0.007	10.8
cellular carbohydrate catabolic process	17	1.2	0.009	14.3
forebrain development	11	0.8	0.011	17.6
vesicle coating	4	0.3	0.011	18
cellular protein catabolic process	21	1.5	0.012	18.8
regulation of neuronal synaptic plasticity	9	0.6	0.014	21.8
regulated secretory pathway	17	1.2	0.018	27.8
endocytosis	23	1.6	0.019	28.5
regulation of exocytosis	8	0.6	0.020	29.4
mRNA metabolic process	25	1.8	0.020	30
protein folding	24	1.7	0.022	31.8
synaptic vesicle exocytosis	7	0.5	0.023	33.9
biogenic amine metabolic process	14	1	0.025	35.6
neurotransmitter secretion	15	1.1	0.026	37
regulation of synapse structure and activity	12	0.8	0.026	37.2
dendrite development	8	0.6	0.027	38
tricarboxylic acid cycle	6	0.4	0.027	38.3
membrane budding	4	0.3	0.028	38.8
dopamine biosynthetic process	4	0.3	0.028	38.8
central nervous system development	30	2.1	0.028	38.8
cell morphogenesis	53	3.7	0.028	39.5
actin filament-based process	25	1.8	0.029	40.2
protein catabolic process	24	1.7	0.033	44.5
amine biosynthetic process	14	1	0.035	46.8
ER to Golgi vesicle-mediated transport	10	0.7	0.037	48.4
protein polymerization	10	0.7	0.037	48.4
synaptogenesis	7	0.5	0.038	49.8
regulation of RNA metabolic process	6	0.4	0.040	50.9
positive regulation of protein catabolic process	3	0.2	0.042	52.6
acetyl-CoA metabolic process	8	0.6	0.046	56.6
translational elongation	6	0.4	0.047	57.2
hexose metabolic process	22	1.5	0.048	58

B

GOTERM_BP_5	Count	%	P-Value	FDR
modification-dependent macromolecule catabolic process	52	2	0.000	0
cellular protein catabolic process	52	2	0.000	0
intracellular transport	156	6	0.000	0
protein catabolic process	58	2.2	0.000	0
protein transport	124	4.8	0.000	0
synaptic vesicle transport	19	0.7	0.000	0.5
cellular carbohydrate catabolic process	30	1.2	0.000	0.5
acetyl-CoA metabolic process	16	0.6	0.000	0.8
cell redox homeostasis	17	0.7	0.000	0.8
cell part morphogenesis	60	2.3	0.000	0.8
cell projection morphogenesis	60	2.3	0.000	0.8
cell projection organization and biogenesis	60	2.3	0.000	0.8
intracellular protein transport	87	3.4	0.000	0.9
secretory pathway	62	2.4	0.001	1.4
exocytosis	31	1.2	0.001	1.7
monosaccharide catabolic process	23	0.9	0.001	1.8
Golgi vesicle transport	28	1.1	0.001	2.4
phosphorylation	128	4.9	0.002	3.6
ER to Golgi vesicle-mediated transport	18	0.7	0.002	4
cytoskeleton-dependent intracellular transport	27	1	0.004	5.9
G1/S transition of mitotic cell cycle	15	0.6	0.004	6
protein depolymerization	13	0.5	0.004	6.4
interphase of mitotic cell cycle	23	0.9	0.004	6.7
protein modification process	223	8.6	0.005	7.6
interphase	23	0.9	0.005	7.9
synaptic transmission	85	3.3	0.006	9.5
regulation of cytoskeleton organization and biogenesis	14	0.5	0.006	10.2
regulation of organelle organization and biogenesis	14	0.5	0.006	10.2
neuron differentiation	64	2.5	0.006	10.5
microtubule-based movement	23	0.9	0.007	10.8
microtubule-based process	43	1.7	0.007	11.2
secretion by cell	66	2.5	0.007	11.4
neuron development	50	1.9	0.007	11.7
cell morphogenesis	93	3.6	0.008	13.1
nerve-nerve synaptic transmission	16	0.6	0.008	13.3
catecholamine metabolic process	13	0.5	0.008	13.6
protein folding	40	1.5	0.009	14.2
tricarboxylic acid cycle	9	0.3	0.009	14.7
coenzyme catabolic process	10	0.4	0.009	15
cellular morphogenesis during differentiation	43	1.7	0.010	16.2
regulation of neurotransmitter levels	35	1.3	0.010	16.8
central nervous system development	51	2	0.011	17.2
neurite morphogenesis	40	1.5	0.012	18.7
mitochondrial transport	14	0.5	0.012	19.1
protein polymerization	16	0.6	0.014	22.5
dopamine metabolic process	10	0.4	0.016	25.1
regulation of RNA metabolic process	9	0.3	0.017	26
mRNA metabolic process	40	1.5	0.019	28.1
small GTPase mediated signal transduction	69	2.7	0.019	28.7
regulation of actin polymerization and/or depolymerization	11	0.4	0.020	29.3
negative regulation of cellular component organization and biogenesis	11	0.4	0.020	29.3
aerobic respiration	10	0.4	0.021	31.3
translational elongation	9	0.3	0.022	32.9
forebrain development	15	0.6	0.023	33.5
vesicle docking	9	0.3	0.029	40.4
Wnt receptor signaling pathway	22	0.8	0.029	40.6
biogenic amine metabolic process	21	0.8	0.029	40.8
RNA transport	11	0.4	0.030	41.6
nucleic acid transport	11	0.4	0.030	41.6
protein targeting to mitochondrion	8	0.3	0.031	42.5
actin filament depolymerization	7	0.3	0.032	43.7
foregut morphogenesis	4	0.2	0.032	43.7
synaptic vesicle exocytosis	9	0.3	0.046	56.3
actin filament-based process	39	1.5	0.047	56.8
