# Supplementary material for: Expression Profiling of a Genetic Animal Model of Depression Reveals Novel Molecular Pathways Underlying Depressive-Like Behaviours
Source: PLoS One. 2010 Sep 7;5(9):e12596. doi: 10.1371/journal.pone.0012596 (PMC2935375; doi:10.1371/journal.pone.0012596)
Supplement: Table S5 — Primer and probe sequence information for the real-time PCR assays. (0.03 MB PDF) [file pone.0012596.s005.pdf]

Gene	Forward Primer Sequence	Reverse Primer Sequence	Probe Sequence (5'FAM, 3'TAMRA)
TMEM176A	TGTGCTGGTACGTGTTGCTAGA	GTCAGGGAACCAGAGAACATTCTG	CCATGACGCAGGTGACCTAACCAGTCC
FAM111A	TGACACTCAAGTCTTGGTCCTTTG	CCCAGCAACATTCCTTCAGC	AATCTCATCAAAAACAGAGTCAAGGAG
CA3	GCTTGCCGGGACTATTGGAC	AGCTCACTGTCATGGGCTCTTTC	AGTAGCAGCCACACAATGCACTCCTCG
Rnase4	TCACGAAGGCATAGTGAGGGTC	ACAGGCAATGACAACTCGCC	CCTACAGTTGTGGGGCACAGAGCTCCC
RGD1565398_predicted	GGTCAAGGCCAGCAACAGC	CGTCGTTGATGAAATCCATGC	TCGAGCGTCTCTTCAGTTCCAGCAGAA
1392736_at	GGCCTCCTGGTTTAGCTGTGT	GGGATGGTGAGAAAACAAGAGAA	AAATGCTGTTTGCCCTCTTCGGTAGACTCT
RICS_predicted	CCTATGTGCCCTCTGGCTTTG	GGATGTGGCAAGGACAGCTC	ATCCACAGAGCAGGACATACGCCACAG
ILF3	CCGAAACATGCTGGGAAGAAG	GGTTGTAAGGTTGCTGCTGGC	ACCCTCCTACAGCTCGGGCTACCAGTC
Pex11b	GGCTTCTTCCTTGGGTCTGC	GAAGAGGCCAGACACAGCATC	TCAGACAGACGCTCCTCTGGTCTTCCA
mss4	GAAGAAGCCGGATCTGGTGG	AAAGCCCACGTTCTCAAAGATG	TCCTCGAGGACATCACCATCAGGATTG
1383058_at	TCAAGGTTGGGCTCCGATTC	AGCGGTGTGGTGTCTCTTGG	CTGCATCTTGGGATCTAGGCCCGTGG
AA859982	CAGAAGCATACTGCTTGTTCCAATG	GGTCATGCCTGCCTTCTGTG	TGTGCATGACTAAACAGTGCCATCTAGC
AI137236	TGTTAGGTGTGCACCAGCAG	GGTGGAGGCGTCCTAGATGG	TTTGGAGTTCAAACTCTTTTGGGTGCA
Chrna7	CTTGTCAGCACAAGCCTCGG	GGCCTCGGAAGCCAATGTAG	CAGTGTGGAGCTGAGTGCAGGTGCTG
Chrm2	CCTACCCAGTTAAGCGGACCAC	TGCCAGAAGAGAATGGCTGG	CTGCGTGGGTCCTTTCCTTCATCCTCT
Htr1a	TGGCTCTGGTCCTGCCTTTC	GGATTGAGCAGGGAGTTGGAG	TTAATTATGGCACCCAACAACGCAGGC
Htr2a	TGACCTCAGCACTCGAGCCA	TGTGGATGGACCGTTGGAAG	AGCCTCCTTCAGCTTCCTCCCTCAGAG
Gabbr2	GCCTCCCATGAGAAGCAAGG	GGACCACAGGATAGGCCAGC	TCCAAGTCCTACATCAGGCATCAAGCTGT
Gabbr3	CATGGGCGGTACATGGGA	GCGAAGACCTCCTCCGTAGG	ACAGAAGCATCCCGCACAAGAAGACG
GAPDH	CAAGGTCATCCATGACAACTTTG	GGGCCATCCACAGTCTTCTG	ACCACAGTCCATGCCATCACTGCCA
PPIA	TTATCTGCACTGCCAAGACTGA	CCACAATGCTCATGCCTTCTTTC	CCAAAGACCACATGCTTGCCATCCA
HPRT	TTGCTCGAGATGTCATGAAGGAG	ATGTAATCCAGCAGGTCAGCAAAG	ATCACATTGTGGCCCTCTGTGTGCTG
ACTB	GAGCTATGAGCTGCCTGACG	AGTTTCATGGATGCCACAGGA	CATCACTATCGGCAATGAGCGGTTCC
MRFAP1	CATCTGGGACTGTGGATGGG	CCAACAAACTCATCAGGCAGG	ACACGGCTAGGACTGATGACCTGGGAG
RGD1310230	CTTGCAGAATGCAGGGAAGG	CAAAGGAGCGTGTGTAGGCG	AGAGTGGAGAGCTGACCTGTCGCTGAC
PHPT1_predicted	TTCCTGATGTGGACATTGATTCAG	TTTCCTTGCACTCCCTCGC	TTCGAGTCCACTTAGCAGAGCCTTCCG
CDIPT	CAAAGGTGGTTGGCTCCTCC	TGGCCAGCCAGGACATAGAG	ATTCCAGTGACCTCAGAGAGGCCTCGA
